# Correction: Proteinopathies and OXPHOS dysfunction in neurodegenerative diseases

**DOI:** 10.1083/JCB.20170917212192017c

**Published:** 2018-01-02

**Authors:** Hibiki Kawamata, Giovanni Manfredi

Vol. 216, No. 12, December 4, 2017. 10.1083/jcb.201709172.

In the section Mitochondrial Aβ and OXPHOS in AD, this review cites one study (Cenini et al., 2016) in a statement that does not correctly represent the findings. Therefore, the authors wish to correct the statement to more accurately reflect the results and conclusions of the article by Cenini et al. The authors apologize for the error and any confusion this may have caused.

The HTML and PDF versions of this article have been corrected. The error remains only in the print version and PDF versions downloaded on or before December 20, 2017.

